# A Study on Decoding Models for the Reconstruction of Hand Trajectories from the Human Magnetoencephalography

**DOI:** 10.1155/2014/176857

**Published:** 2014-06-22

**Authors:** Hong Gi Yeom, Wonjun Hong, Da-Yoon Kang, Chun Kee Chung, June Sic Kim, Sung-Phil Kim

**Affiliations:** ^1^MEG Center, Department of Neurosurgery, Seoul National University Hospital, Seoul 110-744, Republic of Korea; ^2^Interdisciplinary Program in Neuroscience, Seoul National University College of Natural Sciences, Seoul 151-742, Republic of Korea; ^3^The Planet SK Co., Ltd., Seongnam 463-400, Republic of Korea; ^4^School of Design and Human Engineering, Ulsan National Institute of Science and Technology, Ulsan 689-798, Republic of Korea; ^5^Department of Neurosurgery, Seoul National University College of Medicine, Seoul 110-744, Republic of Korea; ^6^Department of Brain & Cognitive Sciences, Seoul National University College of Natural Sciences, Seoul 151-742, Republic of Korea; ^7^Sensory Organ Research Institute, Seoul National University, Seoul 151-742, Republic of Korea

## Abstract

Decoding neural signals into control outputs has been a key to the development of brain-computer interfaces (BCIs). While many studies have identified neural correlates of kinematics or applied advanced machine learning algorithms to improve decoding performance, relatively less attention has been paid to optimal design of decoding models. For generating continuous movements from neural activity, design of decoding models should address how to incorporate movement dynamics into models and how to select a model given specific BCI objectives. Considering nonlinear and independent speed characteristics, we propose a hybrid Kalman filter to decode the hand direction and speed independently. We also investigate changes in performance of different decoding models (the linear and Kalman filters) when they predict reaching movements only or predict both reach and rest. Our offline study on human magnetoencephalography (MEG) during point-to-point arm movements shows that the performance of the linear filter or the Kalman filter is affected by including resting states for training and predicting movements. However, the hybrid Kalman filter consistently outperforms others regardless of movement states. The results demonstrate that better design of decoding models is achieved by incorporating movement dynamics into modeling or selecting a model according to decoding objectives.

## 1. Introduction

Brain-computer interfaces (BCIs) aim to establish an artificial interface between the brain and external systems through which a person can control effectors without physical movements [1–4]. BCIs have been applied to rehabilitation of motor functions lost due to neurological disorders. For instance, a number of studies have demonstrated that patients with tetraplegia could control assistive systems directly using BCIs [5–10]. Also, in conjunction with robotic devices, BCIs have been used to detect motor intentions of stroke patients to develop a self-regulating rehabilitation system [11–14]. Restoration of motor functions particularly in people with paralysis has been mostly investigated using invasive BCIs that harness an ensemble of single-unit spiking activity [5–8]. These BCIs have been designed to generate continuous kinematic parameters such as position, velocity, acceleration, force, and joint angles of limb movements [15–20]. Recently, noninvasive BCIs based on electroencephalography (EEG) or magnetoencephalography (MEG) have also been proposed to predict continuous kinematic parameters of arm movements in humans [21–27].

Inference of continuous kinematic parameters from neural signals requires accurate and reliable neural decoding models [28–33]. Decoding models aim to find a functional mapping between neural representations and one or more kinematic parameters. A number of mathematical models have been employed as decoding models for invasive and noninvasive BCIs, including the linear filter, Kalman filter, point process models, neural networks, and support vector machine, to name a few [34–40]. Many BCI studies on the construction of decoding models have been focused on proposing a state-of-the-art machine learning technique as a new decoding model or simply comparing different decoding models in terms of decoding performance to choose the best one for their applications [40]. In particular, the design of noninvasive BCIs has been generally rather concentrated on finding optimal neural features than optimizing decoding models. This is partly because the noninvasive BCI output space is more likely limited and usually discrete so that the characteristics of the output space seem less important to designing decoding models. However, when a noninvasive BCI is used to produce continuous kinematic states, which create a much sophisticated and dynamic output state space, one may need to be concerned with how to incorporate the characteristics of kinematic parameters into decoding models. In addition, departing from a simple comparison among candidate models followed by the selection of the best, it would be beneficial if we understand more about how individual decoding models work in different circumstances. It can then provide a useful guideline for BCI researchers to choose a decoding model appropriate for their own applications.

In the present study, we investigate whether consideration of hand movement dynamics in the design of decoding models can enhance decoding performance. The investigation is conducted on the human MEG data collected from a noninvasive BCI experiment in which the human subjects performed point-to-point arm reaching movements. We focus especially on the nonlinear characteristics of the hand speed profile and its independence of movement directions. While many BCI studies have typically decoded the hand velocity from neural signals, the hand speed alone could also be decoded from neural signals during point-to-point reaching movements [19, 41, 42]. Hence, we propose to decompose the hand velocity into its speed and direction parameters and decode each parameter independently. While we use the standard Kalman filter for hand direction estimation, due to the nonlinear characteristics of hand speed profiles, we simply augment the Kalman filter by adding a nonlinear filter for hand speed estimation. Then, we investigate whether such hybrid decoding of speed and direction can improve hand trajectory reconstruction from the human MEG signals.

Also, we investigate how decoding models are affected by varying model design factors. Here we examine the effect of choosing movement states on different decoding models. Specifically, we study the effect of two distinct arm movement states: the rest and reach states [43]. Performance of individual decoding models is evaluated for two cases when each model estimates the reach state only or both states together. As for decoding models, we examine the two most widely used filters for kinematic estimation in BCIs—the Kalman filter and linear filter—as well as the hybrid filter newly proposed above.

To assess the performance of decoding continuous hand trajectories, we adopt the evaluation measures used for pointing devices [44]. These measures have also been leveraged to assess neural cursor control performance in the previous BCI studies [7, 8, 45] and may well serve to evaluate reconstructed trajectories of pointing movements. Various performance measures used in this study are expected to collectively provide a richer assessment tool for decoding performance.

## 2. Materials and Methods

### 2.1. Experimental Procedure

**Figure 1:**
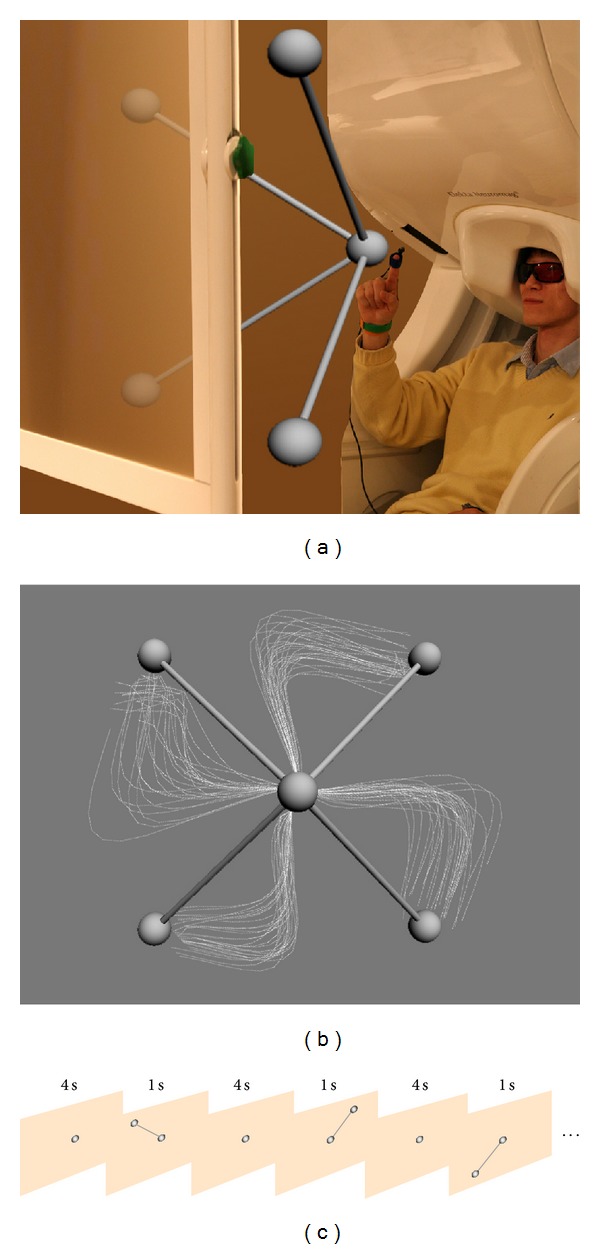
Experiment paradigm. (a) A photograph showing the visual stimuli. Whole-head MEG signals were acquired during point-to-point reaching movements (center-out paradigm). (b) Movement speed profiles for different target directions. Each gray line illustrates a speed profile for each reaching movement towards one of four targets (radial) from the center target (middle). (c) Drawings show the sequence of the visual stimuli. At the beginning of the experiment, a sphere was presented on the center of the screen. After 4 s, a target sphere with a stick connected to the center sphere appeared on one corner for 1 s. The subject was instructed to move his/her right index finger from the center to the target and trace back to the center within this 1 s period. The target appeared in a pseudorandom order.

Nine subjects (19–37 years; five males) participated in the study. All the participants were right-handed (>80 on the Edinburgh Handedness Inventory score) and not color-blind. The institutional review board (IRB) of the Seoul National University Hospital approved this study and all the participants provided written informed consent after the study procedure had been explained to them. During the experiment, the participants were instructed to move their right arm in a specified three-dimensional space while their other body parts were fixed (Figure 1(a)). A cushion was placed under the participants' elbow to minimize the potential artifacts from the arm movement. Participants' head movements were restricted by placing their head in a fixed MEG helmet. In addition, the tSSS filtering was applied to the MEG signals, as described in Section 2.3, to reduce artifacts from external sources. Stereographic images were shown on the screen using the STIM2 system (Neuroscan, El Paso, TX, USA) in order to give instructions of the three-dimensional movements.

In the beginning of the experiment, the participants were shown a sphere in the middle of the screen for four seconds. In this period, the participants were instructed to locate their index finger on the sphere. After this initial period, a target sphere appeared for one second in one of the four corners along with a line that connected the target with the center sphere. During this period, the participants were instructed to move their index finger from the center to the target sphere following the line (a center-out task) and to move back to the center sphere as fast as they could. Average movement time was 930.1 ± 330.5 ms (mean ± SD). A trial ended after this period and continued onto the next initial period of the consecutive trial. No time limit was imposed on a trial because the time interval between trials was large enough (4 s) to allow participants to complete the movement task and rest until the onset of the subsequent movement. The location of target sphere was randomly determined in each of the four corners: upper-left, upper-right, bottom-left, and bottom-right corners. A single session consisted of one hundred twenty trials, including thirty trials per target. Each participant completed two sessions (Figure 1(c)).

### 2.2. Virtual Stimuli

An anaglyph approach was employed to produce three-dimensional images that were used as stimuli in our experiment. An image generated by the anaglyph approach is invisible when a person sees it with the same color of glasses as the image color. The participants wore the colored glasses with a red glass on one eye and a blue glass on the other. Then, if a single object was shown as two different images each being colored in red or blue, each eye would only sense the image with opposite color and the object would stand at the intersection of two visual fields, to create a 3D virtual object [27]. We thus generated two images of the same object with different viewpoints (i.e., different angles) using the Autodesk 3ds MAX 2011 program (Autodesk, San Rafael, CA, USA). Those two images were then converted into an anaglyph image using the Anaglyph Maker software (ver. 1.08; http://www.stereoeye.jp/).

### 2.3. MEG Data Acquisition and Processing

A 306-channel whole-head MEG system (VectorView, Elekta Neuromag Oy, Helsinki, Finland) was used to record human MEG signals during the experiment in the magnetic shielded room. The system consisted of 306 sensors in triplets of two planar gradiometers and one magnetometer distributed at the 102 locations over the whole brain. In this study, only gradiometers were used due to their better signal-noise ratio (SNR) than magnetometers [46]. The MEG signal was digitized at a sample frequency of 600.615 Hz and band-pass filtered at 0.1–200 Hz. A three-axis accelerometer (KXM52, Kionix, NY, USA) was attached on the index finger of the participants to record three-dimensional acceleration signals with the same sample rate as the MEG signals.

We further applied the spatiotemporal signal space separation (tSSS) method to the MEG signals to alleviate external interference noise [47]. The MEG signals were then divided into a series of epochs each from −1 to 2 seconds after target onset. The accelerometer signal was band-pass filtered at 0.2–5 Hz so as to remove linear trends. The index finger velocity was obtained by integrating the accelerometer signal.

We selected sixty-eight gradiometer channels at thirty-four locations over bilateral sensorimotor areas for our study (see [27] for the exact location information). The MEG signal from each of these channels was band-pass filtered at 0.5–8 Hz. The filtered signals were downsampled to 50 Hz. The estimated velocity signals at the *x*-, *y*-, and *z*-coordinates were also downsampled to 50 Hz. In this study, we only used the *x* and *y* velocity signals to simplify decoding analyses because the most variance of the hand trajectories was present in the *x*- and *y*-axis.

### 2.4. Decoding Models

#### 2.4.1. Linear Filter

The *x*- and *y*-coordinates of the hand velocity at time *t* were estimated by a linear filter (LF) that linearly combined the short history of the MEG signals at each of sixty-eight channels to predict the velocity [25, 27]. The size of the history window applied to each channel was 200 ms, corresponding to 11 sample points (a current time point plus 10 preceding time points with a 50 Hz sampling rate). The window size was empirically determined with which the optimal decoding performance was achieved [27]. The prediction by LF was executed for each velocity coordinate as follows:

(1) $$\begin{array}{c} v_{x}\left(t\right)=\overset{n}{\underset{i=1}{\sum }}\overset{m}{\underset{j=0}{\sum }}\alpha_{ij}^{x}z_{i}\left(t-j\right)+\alpha_{0}^{x},\\ v_{y}\left(t\right)=\overset{n}{\underset{i=1}{\sum }}\overset{m}{\underset{j=0}{\sum }}\alpha_{ij}^{y}z_{i}\left(t-j\right)+\alpha_{0}^{y}, \end{array}$$

where *v*_*x*_(*t*) and *v*_*y*_(*t*) are the estimated *x*- and *y*-coordinates of velocity at time *t*, respectively. *z*_*i*_(*t* − *j*) is a MEG signal sample at channel *i* and time *t* − *j*, and *α*_*ij*_^*x*^ and *α*_*ij*_^*y*^ are weights of LF. *α*_0_ is a bias term for each velocity coordinate, *n* is the number of channels (*n* = 68), and *m* is the size of the history window (*m* = 10). The weights, *α*_*ij*_ and *α*_0_, were estimated using the multiple linear regression method.

#### 2.4.2. Kalman Filter

The Kalman filter (KF) has been successfully used as a decoding algorithm of kinematic variables such as position, velocity, and acceleration in a number of BCI studies [5, 7, 8, 34]. Construction of KF is based on linear Gaussian system and observation models as follows:

(2) $$\begin{array}{c} z\left(t\right)=H\left(t\right)d\left(t\right)+\varepsilon \left(t\right), \end{array}$$

(3) $$\begin{array}{c} x\left(t\right)=A\left(t\right)x\left(t-1\right)+\nu \left(t\right). \end{array}$$

The observation model describes how neural observations are generated from movement states (2). *H*(*t*) is the matrix of mapping movement states **x**(*t*) to each neural signal and estimated from the training data by the least-squares method. The observation error vector, **ε**(*t*), is assumed to be a multivariate Gaussian random vector with zero mean and a covariance matrix, *Q*(*t*). Here we assume that *H* and *Q* are time invariant. The system model describes the evolvement of movements in time (3). It is assumed to follow a Markov process. The system matrix *A*(*t*) is also estimated by the least-squares method. The system error vector ***ν***(*t*) is assumed to follow a multivariate Gaussian random vector with zero mean and a covariance matrix of *W*(*t*). Again, we assume that *A* and *W* are time invariant.

Once the model parameters are estimated from the training data, the hand velocity signals (a 2D velocity state in the case of KF) can be decoded by KF following the two steps. In the first step, the system model predicts the velocity state at time *t* from the state at *t* − 1. In the second step, the observation model estimates a neural vector using the predicted velocity state and updates the predicted velocity state based on a difference between those observed and the predicted neural data. These steps are recursively applied to every neural observation.

#### 2.4.3. A Hybrid Kalman Filter

With an aim to incorporate the arm movement dynamics into a modeling scheme, we exploited two particular aspects of the hand speed characteristics, including nonlinearity and independence. That is, the hand speed exhibits a typical bell-shaped nonlinear profile during a point-to-point movement and its profile is independent of movement direction (see Figure 1(b)). To this end, we first added new state variables to the velocity state variables to represent the speed state. In particular, we created three speed state variables including *r*(*t*), *r*(*t* − 1), and *r*(*t* − 2) to represent the states of current speed, the absolute acceleration, and the absolute jerk, respectively. Selection of three speed state variables was based on an observation that the bell-shaped speed profile might be described by at least three temporal terms. Then, the state vector of the new Kalman filter at time *t* was given by

(4) $$\begin{array}{c} x\left(t\right)={\left[r\left(t\right)r\left(t-1\right)r\left(t-2\right)d_{x}\left(t\right)d_{y}\left(t\right)\right]}^{T}, \end{array}$$

where *d*_*x*_(*t*) and *d*_*y*_(*t*) denote *x*- and *y*-direction, respectively.

Next, we augmented the Kalman filter by adding a nonlinear filter. This nonlinear filter predicted the current hand speed from the three speed state variables. We realized the nonlinear filter using a multilayer perceptron (MLP), composed of eighteen hidden units with the hypertangent activation functions and one output unit with the logistic sigmoid activation function. Note that MLP only receives the speed state without direction input to be consistent with our assumption of independence of speed from direction. MLP is trained using the scaled conjugate gradient algorithm.

The hand direction is directly estimated from a subset of the state vector of the Kalman filter, **x**_*v*_(*t*) = [*d*_*x*_(*t*)  *d*_*y*_(*t*)]^*T*^. At every estimation iteration, the direction vector at time *t* is normalized by **x**_*v*_(*t*)/||**x**_*v*_(*t*)|| to have a unit length. The direction vector is multiplied by the estimated speed value from MLP to finally produce a velocity estimate at time *t*.

### 2.5. Performance Evaluation

In order to evaluate decoding performance, we first used a conventional measure using root-mean-squared-error (RMSE) to assess gross accuracy. RMSE measures the grand average of the root of squared errors between the true and decoded hand trajectories. In addition, to assess finer characteristics of continuous hand trajectories decoded by BCIs, we evaluated decoded trajectories using pointing device assessment metrics [44]. A decoded hand trajectory was evaluated with respect to the task axis in terms of four metrics: orthogonal direction change (ODC), movement direction change (MDC), movement error (ME), and movement variability (MV). The task axis was defined as an optimal straight path between the starting point and the center of the targets. ODC measures directional changes orthogonal to the task axis. ODC represents the consistency of a decoded trajectory toward the target. MDC measures directional changes in parallel with the task axis. MDC depicts the smoothness of the decoded trajectory. ME measures a mean distance of the decoded trajectory from the task axis. ME exhibits how much a decoded trajectory is different from the optimal path. MV measures the standard deviation between a decoded trajectory and the task axis. MV depicts the straightness of a decoded trajectory. For more details of these four metrics, the reader can be referred to MacKenzie et al. [44].

## 3. Results

**Figure 2:**
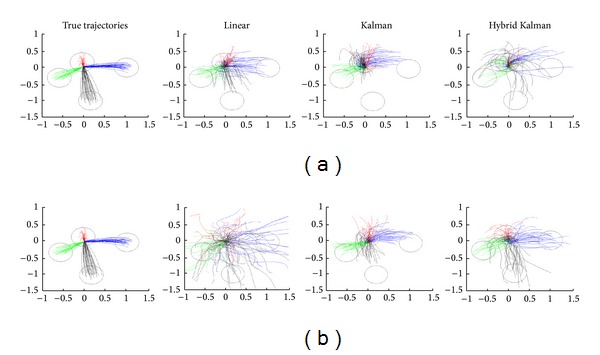
Reconstructed 2D hand trajectories by three different algorithms (LF, Kalman, and hybrid Kalman). Each line shows a single trial movement. Different colors indicate reaching movements towards different targets. Circles illustrate a target area. (a) Reconstruction results for both reach and rest. (b) Reconstruction results for reach only.

Using either the reaching only movement data or the reaching and resting movement data, along with the corresponding multichannel MEG data, we trained three different decoding models and reconstructed the hand trajectories.  Figure 2 illustrates a sample of the true and reconstructed hand trajectories by each decoding model in the 2D space in one of the participants. Specifically, Figure 2(a) shows the reconstruction of reaching movements after training and estimating both reaching and resting movements, whereas Figure 2(b) shows reaching movements after training and estimating reaching movement only. The reconstructed hand trajectories followed similar paths to the true trajectories in most trials.

**Figure 3:**
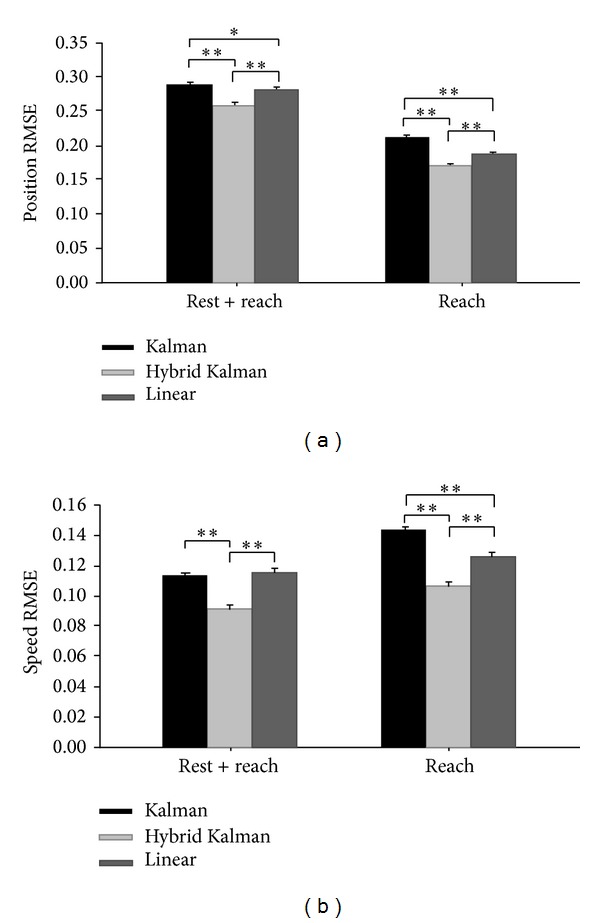
The average RMSE between the true and reconstructed hand trajectories decoded by different algorithms (linear, Kalman, and hybrid Kalman filters). (a) The average RMSE of the hand position. Error bars indicate the standard errors of the means. *P < 0.05; **P < 0.01. (b) The average RMSE of speed.

We first evaluated the gross performance using RMSE. We evaluated RMSE of the hand speed as well as the hand position, because we aimed at the improvement of speed decoding. The RMSE measurement revealed that the proposed hybrid Kalman filter produced significantly lower errors than the standard Kalman filter or the linear filter (paired *t*-test, *P* < 0.01) for both position and speed (Figure 3). Such lower errors were achieved regardless of movement states: reach and rest or reach only. The linear filter exhibited lower position and speed prediction errors than the Kalman filter when the data of reaching movement were only used (*P* < 0.01). However, the performance of two filters was on par with each other when the data of both reaching and resting movements were used. Notice that overall RMSE of the position decreased when the reaching movement data were only used (Figure 3(a)), whereas overall RMSE of the speed increased for the same case (Figure 3(b)).

Next, we evaluated fine measures including ODC, MDC, ME, and MV for individual trajectories (see Methods for details of each measure). The evaluation results are summarized in Table 1. The hybrid Kalman filter produced the fewest ODC and MDC for both cases of reach and rest or reach only. The linear filter and the Kalman filter, on the other hand, produced more ODC and MDC, showing that the trajectories by these filters were relatively less consistent and smooth. The hybrid Kalman filter reduced ODC and MDS compared to the linear filter by approximately 55% and 51%, respectively. On the contrary, the standard Kalman filter produced the lowest ME and MV compared to the hybrid Kalman filter and the linear filter. The hybrid Kalman filter produced lower ME and MV than the linear filter when the data of both reach and rest were used but higher than the linear filter when the data of reach only were used. The Kalman filter reduced ME and MV compared to the linear filter by approximately 27% and 31%, respectively. Note that the four measures of the linear and Kalman filters were reduced when the reaching movement data was used only (all measures; *P* < 0.01), while those of the hybrid Kalman filter remained relatively steady (ODC; *P* = 0.828, MDC; *P* = 0.022, ME; *P* = 0.204, MV; *P* = 0.065).

## 4. Conclusions and Discussion

The present study addressed how we could improve the design of a decoding model in an MEG-based noninvasive BCI by incorporating the properties of continuous arm movements. Based on the fact that the hand speed shows nonlinear profiles and is generally independent of movement direction, we designed a model that separately decoded speed and direction to reconstruct hand trajectories from the human MEG. The model was built by adding a nonlinear filter for speed decoding to the Kalman filter while the direction information was directly inferred by the Kalman filter. We demonstrated that this hybrid Kalman filter generated lower prediction errors to reconstruct the hand trajectory and also to estimate the hand speed than the standard Kalman filter and the linear filter. We also investigated how the selection of movement states affected decoding performance. We found that the linear filter performed better than the Kalman filter when the data of reaching movement was only used. On the other hand, the performance of the two filters was similar when the data of both reaching and resting movements was used. This result demonstrates that the choice of a decoding model may be dependent on the type of continuous movements a BCI is designed to estimate.

We note that speed RMSE increased but position RMSE decreased when we used the data of reaching movements only. It may imply that speed decoding could be improved by training more diverse movements including reach and rest. However, position RMSE could be improved by training more specific movements including reach only and allowing decoding models to focus on movement prediction. We also note that the linear filter outperformed the Kalman filter when the data of reaching movements was only considered. This may imply that, for stereotyped movements, the simple direct decoding approach such as the linear filter could perform reasonably well and the generative decoding approach such as the Kalman filter might provide little advantage. However, when we modified the Kalman filter to fit to the characteristics of BCI output (here, continuous arm movements), we could significantly improve decoding accuracy.

We used a variety of assessment tools to evaluate BCI performance. The fine measures adopted in this study, including ODC, MDC, ME, and MV, allowed us to look into more details of how accurately and reliably the hand trajectories were reconstructed [7, 8, 45]. In fact, four different measures revealed certain advantages and disadvantages of using the new hybrid Kalman filter, demonstrating that a new decoding model should be evaluated in multiple angles. This would not be possible if we only used a gross measure of RMSE. The worse outcomes in terms of ME and MV with the hybrid Kalman filter might be due to its wide range of variability in the reconstructed trajectories. It shows the current limitations of the proposed model and also gives us a direction of how to improve this model to improve the straightness of the trajectory in the following study.

Finally, we would like to underline that the present study demonstrates an approach of improving neural decoding models, not by adopting a cutting-edge machine learning algorithm but by taking the properties of a BCI output into account. Demonstration of decoding improvement by redesigning a current Kalman decoding model based on hand movement characteristics may indicate the importance of design factors in decoding models and thus in BCIs. We also examined a possibility of estimating the hand speed directly from the KF state variable without adding a nonlinear filter to the state variables. It resulted in a decoding performance significantly worse than the performance of using an MLP (*P* < 0.01). Hence, we verified that adding a nonlinear filter improved performance further. Yet, we also fully recognize that a complete evaluation of a decoding model should be done in a closed-loop BCI system, and therefore we will pursue online BCI studies using our new approach in the future.

## Figures and Tables

**Table 1:** Movement prediction performance by different algorithms.

Algorithm	Movements	ODC	MDC	ME	MV
Kalman	Reach + rest	19.728 ± 0.227	23.262 ± 0.242	0.148 ± 0.003	0.190 ± 0.003
Hybrid	Reach + rest	8.015 ± 0.175	9.321 ± 0.180	0.191 ± 0.004	0.251 ± 0.005
Linear	Reach + rest	20.010 ± 0.277	21.585 ± 0.333	0.215 ± 0.004	0.300 ± 0.006
Kalman	Reach	12.995 ± 0.167	16.992 ± 0.181	0.125 ± 0.001	0.169 ± 0.002
Hybrid	Reach	7.956 ± 0.113	9.841 ± 0.153	0.188 ± 0.002	0.258 ± 0.002
Linear	Reach	15.410 ± 0.193	17.441 ± 0.244	0.159 ± 0.002	0.222 ± 0.003

All values are the mean ± standard error of the mean. ODC: orthogonal direction changes; MDC: movement direction changes; ME: movement error; MV: movement variability.
